# Immediate Effects of Whole-Body Vibration Associated with Squatting Exercises on Hemodynamic Parameters in Sarcopenic Older People: A Randomized Controlled Trial

**DOI:** 10.3390/ijerph182211852

**Published:** 2021-11-12

**Authors:** Fabiana Angélica de Paula, Vanessa Amaral Mendonça, Vanessa Kelly da Silva Lage, Guilherme Pinto da Silva, Hellen Cristina de Almeida, Liliana Pereira Lima, Joyce Noelly Vítor Santos, Daniela Pereira de Castro, Camila Franciele da Paixão, Ana Luiza da Silva Nunes Teixeira Rodrigues, Vinícius Cunha de Oliveira, Pedro Henrique Scheidt Figueiredo, Mario Bernardo-Filho, Ana Cristina Rodrigues Lacerda, Redha Taiar

**Affiliations:** 1 Programa de Pós-Graduação Multicêntrico em Ciências Fisiológicas, Laboratório de Inflamação e Metabolismo, Universidade Federal dos Vales do Jequitinhonha e Mucuri, Diamantina 39100-000, Brazil; fabianaangelicadepaula@yahoo.com.br (F.A.d.P.); vanessakellysl@hotmail.com (V.K.d.S.L.); hellen.nut@gmail.com (H.C.d.A.); liliplima@hotmail.com (L.P.L.); 2 Programa de Pós-Graduação Multicêntrico em Ciências Fisiológicas, Programa de Pós-Graduação em Reabilitação e Desempenho Funcional, Programa de Pós-Graduação em Ciências da Saúde, Laboratório de Inflamação e Metabolismo, Universidade Federal dos Vales do Jequitinhonha e Mucuri, Diamantina 39100-000, Brazil; vaafisio@hotmail.com; 3 Programa de Pós-Graduação em Reabilitação e Desempenho Funcional, Universidade Federal dos Vales do Jequitinhonha e Mucuri, Diamantina 39100-000, Brazil; guilhermeps_ufvjm@hotmail.com (G.P.d.S.); joyce_noelly@outlook.com (J.N.V.S.); vcunhaoliveira@gmail.com (V.C.d.O.); phsfig@yahoo.com.br (P.H.S.F.); 4 Laboratório de Inflamação e Metabolismo, Universidade Federal dos Vales do Jequitinhonha e Mucuri, Diamantina 39100-000, Brazil; castrodanip@gmail.com (D.P.d.C.); kmilladtna@yahoo.com.br (C.F.d.P.); izarodrigues97@hotmail.com (A.L.d.S.N.T.R.); 5 Laboratório de Vibrações Mecânicas e Práticas Integrativas, Departamento de Biofísica e Biometria, Instituto de Biologia Roberto Alcântara Gomes and Policlínica Américo Piquet Carneiro, Universidade do Estado do Rio de Janeiro, Rio de Janeiro 20950-000, Brazil; bernardofilhom@gmail.com; 6 Programa de Pós-Graduação Multicêntrico em Ciências Fisiológicas, Programa de Pós-Graduação em Reabilitação e Desempenho Funcional, Programa de Pós-Graduação em Ciências da Saúde, Laboratório de Fisiologia do Exercício, Universidade Federal dos Vales do Jequitinhonha e Mucuri, Diamantina 39100-000, Brazil; lacerdaacr@gmail.com; 7 MATIM, Department of Sport Science, Université de Reims, CEDEX 2, 51687 Reims, France

**Keywords:** sarcopenia, older people, whole-body vibration, physical exercise, hemodynamic responses

## Abstract

Whole-body vibration (WBV) exercises have recently been introduced as a nonpharmacological therapeutic strategy for sarcopenic older people. The present study aimed to evaluate the effect of WBV exercise on hemodynamic parameters in sarcopenic older people. Forty older people, divided into groups of nonsarcopenic (NSG = 20) and sarcopenic (SG = 20), participated in the study and were cross randomized into two interventions of eight sets of 40 s each, these being squatting with WBV and squatting without WBV. Heart rate (HR), peak heart rate (peak HR), systolic blood pressure (SBP), diastolic blood pressure (DBP), double product (DP), mean arterial pressure (MAP), and subjective perception of effort (SPE), were assessed at baseline, during, and after a single WBV session. The HR, peak HR, and DP variables were similar at baseline between groups. WBV exercise increased all the hemodynamic parameters both during and immediately after the intervention, in both groups (SG and NSG). The MAP values were similar at baseline between groups; however, in the NSG there was a significant increase during and immediately after the squatting with WBV intervention (*p* < 0.05). The HR behavior, in both groups, showed that there was an increase in HR after the first set of exercises with vibration and this increase was maintained until the final set. The absence of adverse effects of WBV exercise on the cardiovascular system and fatigue suggests this exercise modality is adequate and safe for sarcopenic older people.

## 1. Introduction

Sarcopenia is a progressive and generalized disease of skeletal muscle, recognized in ICD-10 (International Code of Diseases), which is associated with a greater likelihood of adverse outcomes, including falls, fractures, physical disability, mortality [1], and higher health costs [2]. The incidence of sarcopenia increases with age [3], and depending on the environment [4], can affect up to 33% of the population of older people living in the community [5], and up to 68% of individuals living in long-term care institutions [6].

The progressive loss of skeletal muscle mass occurring with aging may be associated with an inadequate supply of blood flow to the skeletal muscle. This plays an important role in the development of sarcopenia, as demonstrated in a study in which the blood pressure (BP) variability index was significantly higher at rest in both male and female participants in the group with the lowest appendicular skeletal muscle mass (ASM) [7]. Studies show that aging attenuates coronary blood flow and myocardial perfusion and predisposes older people to adverse cardiac events [8,9]. Moreover, aortic diastolic blood pressure (DBP) response to muscle metaboreflex activation is attenuated in older people with dynapenia but positively affects walking performance in nondynapenic older people [10]. Therefore, hemodynamic changes play an important role in the development of sarcopenia [7].

Thus, sarcopenia can be considered one of the most important causes of reduced cardiorespiratory fitness in older people, especially the frail [11,12]. Moreover, it is associated with cardiovascular risk factors such as altered endothelial function, reduced exercise tolerance, effects on BP, and reduced heart-rate recovery in sarcopenic older people with heart failure [13].

Physical exercise, nutrition, hormone replacement, and lifestyle interventions are among the forms of treatment for sarcopenia [14,15,16]. Interventions that promote a physically active lifestyle, reduced sedentary behavior and increased energy expenditure are recommended for the treatment and prevention of sarcopenia [17,18]. Although traditional resistance training is often recommended, evidence indicates that the effects of resistance exercise can be optimized if combined with whole-body vibration (WBV) [19]. A study noted improvement in muscle power by adding vibration therapy to conventional resistance exercise [20]. WBV has been introduced as a complementary and viable form of exercise for frail older people [21,22,23], and especially for sarcopenic older people [24,25].

The physiological effects of WBV are explained by greater muscle activation, leading to better cardiorespiratory responses and muscle activity during exercise [20,26,27,28]. Some of these immediate physiological effects, such as increased heart rate (HR) during WBV, are supported by studies in sedentary older people [26,28], in older people with chronic obstructive pulmonary disease (COPD) [29], and in maintenance of cardiovascular responses in older people with metabolic syndrome [30].

Currently, there is no clear consensus on clinical intervention for sarcopenia [25,31]. Furthermore, the majority of sarcopenic older people have a sedentary lifestyle and are frequently reluctant to start a conventional exercise program. WBV may represent a form of well-tolerated exercise for this population. However, there are no studies evaluating the immediate effects of WBV on hemodynamic responses in sarcopenic older people.

Thus, this study aimed to evaluate the effects of one session of squatting exercises with and without WBV on hemodynamic parameters in sarcopenic older people. The hypothesis is that a single WBV session promotes changes in hemodynamic parameters in individuals with sarcopenia and that there is a difference between the squatting exercise with and without WBV.

## 2. Materials and Methods

### 2.1. Participants and Sample Size Estimation

A crossover randomized controlled trial was conducted between February 2018 and January 2020 with older people living in the community. Participants were recruited through verbal invitation, leaflets, and visits to basic health units and physicians’ offices, or through communication (internet, radio). The inclusion criteria were people aged 60 or over of either sex, who met the criteria of sarcopenia diagnosis, according to relative skeletal muscle index (RSMI) cutoff points described by the European Working Group Consensus on Sarcopenia in Older People—EWGSOP [1]. Exclusion criteria were (1) presence of acute illness; (2) decompensated chronic disease; (3) taking Beta-blocker medication; (4) participation in any physical training program three months prior to the beginning of the assessments; (5) contraindication to the vibrating platform, such as epilepsy, gallstones or kidney stones, neuromuscular and neurodegenerative diseases, stroke, serious heart disease, or those with an implant, bypass, or stent; and (6) cognitive impairment.

The required sample size was determined according to the study by Ribeiro et al. [32]. Considering an effect size of 0.47, α = 0.05, and power of 0.94, found by two-way ANOVA, the estimated sample size was 18 individuals per group (non-sarcopenic and sarcopenic). The value obtained was increased by 11% to suppress potential losses during the execution of the project, with 20 individuals per group, totaling 40 participants. The distribution of participants between the groups was controlled by sex, age, and drug class, to minimize the influence of confounding variables.

### 2.2. Diagnostic Criteria to Sarcopenia

Participants were evaluated using dual-energy X-ray absorptiometry (DXA) (Lunar, DPX, Madison, WI, USA) for body composition measurements, and for the diagnosis of sarcopenia, relative skeletal muscle index (RSMI) cutoff points were considered using Appendicular skeletal muscle mass (ASM) divided by height squared. The cutoff point for the diagnosis of sarcopenia was <7.0 kg/m^2^ for men and <5.5 kg/m^2^ for women [1,33].

### 2.3. Procedures

#### 2.3.1. Body Mass Index (BMI)

BMI was calculated by dividing body mass (kg) by the square of height (meters), adopting the cutoff point for eutrophic BMI between 22 kg/m^2^ and 27 kg/m^2^ [34].

#### 2.3.2. Body Composition Assessment

Total body mass, fat mass, and lean mass were assessed using DXA. Fat mass and lean mass were assessed through total body analysis and by body segment (upper, lower, and arms and legs).

#### 2.3.3. Functionality Assessment

To assess functionality, the following tests were performed: walking speed over 4 m, where the cutoff point used was ≤0.8 m/s [1]; Short Physical Performance Battery (SPPB), which is a functional performance test composed of standing static and balancing in three positions (side-by-side stands, semi-tandem and tandem); 4 m gait speed test; and 5STS (timed test to sit and stand up from a chair without arms five times) [35]. The cutoff point in SPPB is ≤8 [1] for a diagnosis suggestive of functional impairment. In addition, handgrip strength (HS) was also measured, being assessed using the Jamar^®^ dynamometer, with cutoff points for sarcopenia for low handgrip strength being <27 kgf and <16 kgf for men and women, respectively [1]. The assessors were blinded in relation to the groups in which the participants were included.

### 2.4. Interventions

All the experimental procedures were conducted in the same place and on a set schedule. Participants were stratified according to the diagnosis of sarcopenia into the nonsarcopenic group (NSG) and sarcopenic group (SG). The order of execution of the two experimental situations was randomized through a simple draw, in which the protocols were marked by numbers, whereby number 1 corresponded to squatting with WBV and number 2 to squatting without WBV. The participants were randomly allocated to one of the protocols, and after a washout period of one week, they performed the other intervention. All participants performed the intervention protocol (WBV stimulus) and the control (squat without WBV).

#### 2.4.1. Exercise Intervention with WBV

The vibration exposure consisted of performing dynamic squatting exercises (8 sets of 40 s) with a vibration stimulus (frequency of 40 Hz and amplitude of 4 mm) performed on a commercial model of a vibration platform (VP) (FitVibe^®^, GymnaUniphy NV, Bilzen, Belgium) [26]. This vibration frequency and amplitude were selected due to prototype renders of an acceleration range of 2–5 g [27]. The participants were instructed to perform 3 s of isometric flexion of 60° and 3 s of isometric flexion of the knees at 10°. Between the sets, the participants were instructed to remain at rest for 40 s in the orthostatic position on the turned-off VP. The 60° angle was measured for each volunteer using a universal goniometer before initiating the exercise sets. A barrier was placed at the gluteal region to limit the flexion degree of the knees [32,36]. The exercise execution time was around 10 min.

#### 2.4.2. Exercise Intervention without WBV

The without-WBV intervention was performed with the same dynamic squatting exercises (8 sets of 40 s) with the VP turned off. The participants were instructed to perform 3 s of isometric flexion of 60° and 3 s of isometric flexion of the knees at 10°. Between the sets, the participants were instructed to remain at rest for 40 s in the orthostatic position on the turned-off VP. The 60° angle was measured for each volunteer using a universal goniometer before initiating the exercise sets. A barrier was placed at the gluteal region to limit the flexion degree of the knees [32,36]. The exercise execution time was around 10 min.

### 2.5. Data Collection

#### 2.5.1. Hemodynamic Measurements

At baseline, during, and in the first minute after exercise, systolic blood pressure (SBP), diastolic blood pressure (DBP), HR, and peak HR were measured. Double product (DP) was obtained by multiplying SBP and HR [37,38]. The values of the SBP and DBP measurements were used to calculate mean arterial pressure (MAP) [39]. The percentage of maximum heart rate (HRmax) predicted for age was calculated according to the formula (HRmax = 220 − age) [40].

#### 2.5.2. Subjective Perception of Effort (SPE)

At the beginning and immediately after the end of each exercise session, participants assigned a value to the SPE on the modified Borg scale. This is a scale from 0 to 10, which represents a linear increase with exercise intensity, where zero means no fatigue and 10 is maximum fatigue. [41].

### 2.6. Statistical Analysis

Data were analyzed using SPSS version 22.0 and GraphPad Prism 7.0. The data normality was verified using the Shapiro–Wilk test. Descriptive analysis was expressed as mean, standard deviation, and a 95% confidence interval (95% CI). Between-group comparisons for baseline data were performed through unpaired t-test or Mann–Whitney test, as appropriate. Within-group and between-group differences on hemodynamics postintervention outcomes were analyzed using two-way ANOVA with Bonferroni post hoc test. The power and effect size were calculated using Gpower 3.1 software. The level of statistical significance was set at 5%.

## 3. Results

**Figure 1:**
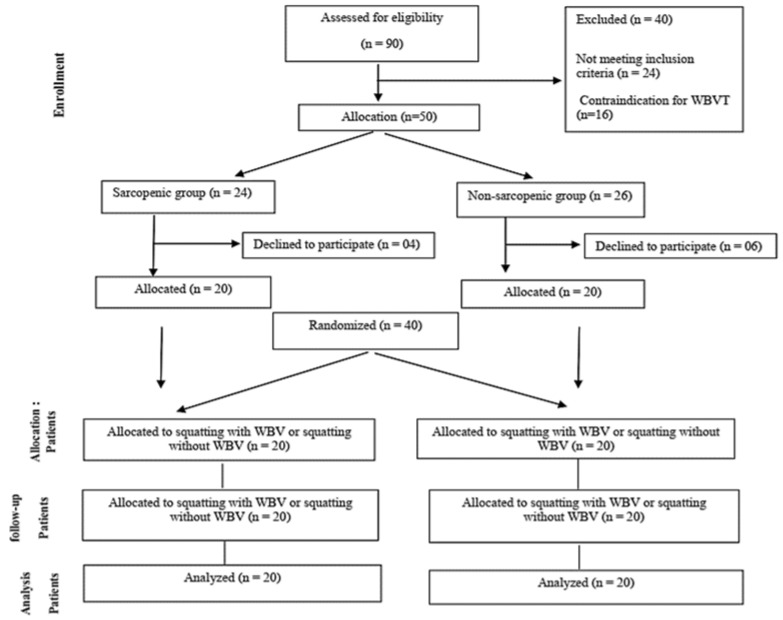
Flowchart of the participants.

All 40 participants completed the study and were included in the analysis as allocated (intention-to-treat analysis). The same participant went through both protocols, in a randomized and crossed way (Figure 1).

### 3.1. Characteristics of the Participants

Forty older people, 20 women and 20 men, participated in the study. As expected, there were significant differences in body composition variables. The SG had a lower fat mass when compared to the NSG (*p* < 0.01). As for RSMI, when stratified by sex, men and women in the SG had lower RSMI when compared with those in the NSG (*p* < 0.01). The SG showed worse performance on the Sit-to-Stand test (5STStest) (*p* = 0.01) when compared to the NSG. In both groups, 45% of participants were taking antihypertensives, and 50% and 55% of nonsarcopenic and sarcopenic participants, respectively, were taking no medication (Table 1).

### 3.2. Hemodynamic Responses in the Situations with and without WBV in NSG and SG

**Figure 2:**
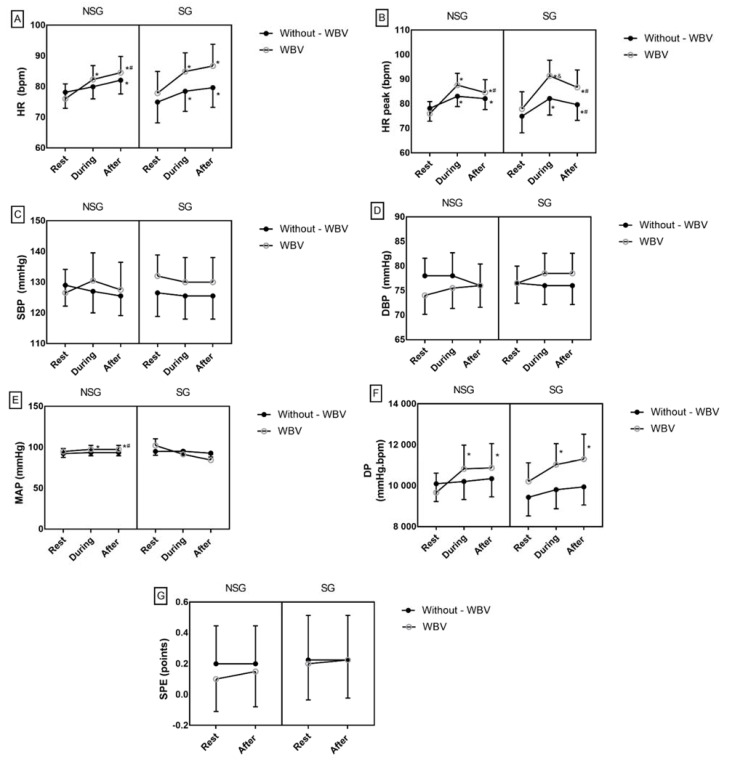
Immediate hemodynamic responses in the interventions with and without whole-body vibration in nonsarcopenic and sarcopenic groups. Data are means and standard deviation. Two-way ANOVA for repeated measures and post hoc Bonferroni. Significance * difference between “rest”; ^#^ difference between “during”, and ^&^ difference between WBV vs. without WBV (*p* < 0.05). Difference between the mean values at rest between groups (a # b). (**A**)—HR: heart rate; (**B**)—peak HR; (**C**)—Systolic Blood Pressure (SBP); (**D**)—Diastolic Blood Pressure (DBP); (**E**)—MAP: mean arterial pressure; (**F**)—DP: double product; (**G**)—SPE: subjective perception of effort.

The HR (Figure 2A), peak HR (Figure 2B), and DP (Figure 2F) variables were similar at baseline between the groups. WBV exercise increased all the hemodynamic parameters (during and immediately after) in both groups (SG and NSG). There were no differences for SBP (Figure 2C), DBP (Figure 2D), and SPE (Figure 2G) for both groups in squatting exercise interventions with and without WBV. The MAP was similar at baseline between groups; however, in the NSG there was a significant increase during and immediately after the squatting exercises with WBV (*p* < 0.05) (Figure 2E).

### 3.3. Heart Rate (HR) Behavior after Each Set of Exercises in Interventions with and without WBV in NSG and SG

**Figure 3:**
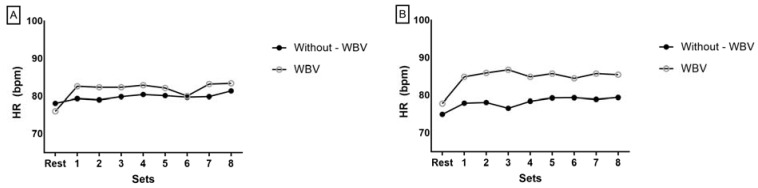
Heart rate (HR) behavior after each set of exercises in situations with and without whole-body vibration in the nonsarcopenic (**A**) and sarcopenic (**B**) groups.

The visual analysis of the HR behavior graph demonstrates that in the NSG, the HR value increased after the first set of squatting exercises with WBV, and this behavior was generally maintained until the last series of the exercise session (Figure 3A). Only a slight variation was observed in the sixth set of exercises, with a drop in the HR value.

In the SG, it was observed that the HR value during pre-exercise (rest) was lower, and from the first set of squatting exercises with WBV there was an increase which remained without a further fall until the final set of exercises (Figure 3B).

The HR values in the squatting exercises without WBV in both groups remained lower compared to with squatting exercises with WBV, and few variations were observed (Figure 3A,B).

### 3.4. Comparison of Hemodynamic Parameters between the Interventions in the NSG and the SG

To compare changes in hemodynamic variables in response to exercise, delta comparisons (during–rest) were performed, as indicated by Δ. There were no differences at baseline between the NSG and the SG for hemodynamic parameters (HR, peak HR, SBP, DBP, MAP, and DP); however, the addition of WBV promoted a significant increase in peak HR in both groups (*p* < 0.000) when compared with the squatting exercise without WBV. In the NSG, the addition of vibration to the squatting exercise promoted a significant increase in HR (*p* < 0.003), MAP (*p* < 0.005), and DP (*p* < 0.003) when compared with the squatting exercise without vibration (Table 2 and Table 3).

## 4. Discussion

To the best of our knowledge, this is the first study to investigate the immediate effects of a WBV session on hemodynamic parameters in the context of sarcopenia. This study demonstrated that the addition of WBV promoted greater variations in hemodynamic variables compared to the squatting exercise alone. However, both groups had the same behavior, proving that it is a safe procedure for individuals with sarcopenia.

As expected, individuals with sarcopenia had low values for anthropometric and body composition variables, which is a similar result to findings from other studies in different populations [42,43], as sarcopenic individuals generally present worse lower-limb muscle performance [44,45]. Despite the differences found, the results of functional tests are within normal cutoff values (EWGSOP) [1].

For the execution of the WBV exercise, a frequency of 40 Hz combined with an intermittent duration close to 360 s per session was used. The choice of the vibration parameters was in line with effective parameters to improve and preserve physical performance in older people with sarcopenia [46,47]. Moreover, the parameters are preferable for stimulating muscles while limiting fatigue [27].

The results showed that one exercise session with WBV was able to increase the mean HR of individuals with sarcopenia and nonsarcopenic individuals. Nonetheless, the addition of vibration promoted a greater variation in HR when compared to the dynamic squatting exercise alone. Similar behavior was observed for peak HR, mainly during the vibration exercise. The findings of this study corroborate the studies of Avelar et al. [26] and Cochrane et al. [27], in which a significant increase in HR was found during WBV exercise compared with exercise without vibration in sedentary older people.

These cardiovascular responses seem to be the result of adding vibration, which induces changes in tissues leading to the activation of muscle spindles, causing a reflex contraction to modulate the stiffness of the muscles involved known as the tonic vibration reflex [48]. This promotes an increase in muscle perfusion associated with peripheral vasodilation where muscle activation occurs, especially in lower limbs. Moreover, the increase in ejection volume due to the increase in venous return is a factor that may be related to the regulation of acute adaptations to exercise, such as the increase in cardiac output and the consequent increase in HR [49,50].

In dynamic exercises, with a greater volumetric load in the left ventricle, the cardiac and hemodynamic responses are proportional to the intensity and muscle mass involved in the activity [51]. Our data demonstrated that WBV was performed at an intensity corresponding to 57% and 59% of the maximum heart rate (HRmax) predicted for age in sarcopenic older people, for mean HR and peak HR, respectively. These values were close to those recommended by the *American College of Sports Medicine* (ACMS) [52] and the *American Heart Association* [53], which consider around 60% of HRmax sufficient to promote cardiovascular physiological changes in sedentary older people [54]. Although exposure to vibration increased the mean HR and peak HR in both groups, the intensity used was mild (light exertion intensity, 57–63% of HRmax predicted for age) [52]. These results are in agreement with a study by Ribeiro et al. [32] that used the same exercise protocol proposed in the present study, in which the WBV, even at low intensity, promoted similar cardiorespiratory changes in healthy people and those with fibromyalgia. According to Licurci et al. [55], older people have a higher risk of developing cardiovascular diseases. As demonstrated in their study, a single session of WBV promoted an improvement in heart rate variability. Moreover, they concluded that WBV does not require physical effort, which makes it potentially beneficial for this population.

In addition to peak HR, SPE and DP are important parameters for monitoring intensity in different exercise modalities [56]. Although differences were observed in hemodynamic variables in the present study, these differences did not cause an increase in SPE, whereby individuals classified the WBV exercise as mild and the vibration did not cause fatigue. Studies have shown that, in addition to exercise intensity, SPE may reflect sensitivity to fatigue related to active musculature during exercise [57]. The addition of WBV increased DP, nevertheless the maximum value for DP during the vibration exercise did not exceed the myocardial ischemia threshold, which is above 30,000 mmHg·bpm and is considered as the cutoff point for angina pectoris [58], showing that the acute exercise protocol with WBV was of low cardiac risk for sarcopenic older people. Moreover, the HR behavior observed in our study during WBV reinforces the safety of the vibration exercise. All patients completed the WBV protocol without adverse effects during the intervention. A study by Aoyama et al. [59] demonstrated that older people with cardiovascular diseases did not present adverse events after an acute session of WBV.

In our findings, the addition of vibration did not promote significant changes in SBP and DBP in either group when compared to the squatting exercise without WBV. In addition to the low exercise intensity, in each NSG and SG, 45% of the participants were hypertensive, being equally distributed in the two groups, which may help to explain the absence of differences in these hemodynamic variables [10]. This result is clinically important because SBP and DBP are determinants of ventricular load and myocardial perfusion pressure, respectively, thus they are more relevant predictors of cardiovascular events [60].

In the NSG, there was a significant increase in MAP during and immediately after vibration exercise. This increase in MAP can be explained by the vibration, which increases muscle stimulation [61], associated with the dynamic squatting exercise, and can potentialize the effect and significantly stimulate the cardiovascular system, increasing HR, blood flow [62], and blood volume [63,64]. These increases may be related to the contraction–relaxation reflex stimulated by vibration, causing greater muscle mass to be recruited (such as the trunk muscles) to allow the individual to continue exercising [65]. The attenuated MAP responses in the SG can be explained as a function of low muscle mass, inadequate supply of blood flow, and, consequently, low capillarization of the skeletal muscle present in sarcopenia [7].

Despite the promising results, the present study has some limitations. We acknowledged that despite being sarcopenic, the older people participating in the study were living in the community and were independent, which may have impacted the severity of the disease, and thus, the magnitude of the physical functions and the effects of the interventions. Therefore, further studies involving a spectrum of sarcopenia-severity patients are warranted. Although the number of participants was based on sample size calculation, the low sample size may have influenced the absence of significant statistical differences for some variables and limited the possibility for extrapolations from the current findings.

The strengths and applications of the findings of this study are that the effects during and immediately after a short session of low-intensity WBV exercise induces safe changes in hemodynamic parameters in sarcopenic older people. Moreover, it showed good adherence from the study participants, and its application in sarcopenic older people who are reluctant to practice physical exercise may be of interest.

## 5. Conclusions

WBV exercise induces safe changes in hemodynamic parameters in sarcopenic older people. The absence of adverse effects of WBV exercise on the cardiovascular system and fatigue shows that this exercise modality can be considered for sarcopenic older people. However, the long-term effects of the WBV need to be studied in this population.

## Figures and Tables

**Table 1:** Clinical, demographic and functional characteristics of participants at baseline.

Characteristics	NSG (*n* = 20)	SG (*n* = 20)	*p*-Value
Demographic and body composition			
Age (years)	72.4 (69.1–75.8)	71.6 (67.7–75.5)	0.73
Sex (Men/Women)	11/9	11/9	
BMI (kg/m^2^)	24.9 (23.7–26.2)	21.2 (20.2–22.2)	**0.01**
BF (%)	32.8 (29.8–35.8)	31.7 (27.8–35.7)	0.65
Lean mass (kg)	39.4.1 (35.9–42.8)	33.4 (30.1 -36.6)	**0.01**
Fat mass (kg)	19.1 (17.2–20.9)	15.5 (13.4–17.7)	**0.01**
RSMI Men	8.0 (7.5–8.6)	6.5 (6.1–6.9)	**0.01**
RSMI Women	6.4 (5.9–6.7)	5.2 (5.0–5.4)	**0.01**
Strength and functional tests			
*SPPB* (points)	10.7 (10.3–11.2)	10.1 (9.5–10.8)	0.21
5STS (s)	9.1 (8.3–9.8)	10.9 (9.8–12.1)	**0.01**
Walking speed (m/s)	1.9 (1.7–2.0)	1.7 (1.5–1.8)	0.06
Handgrip strength (kgf)	34.8 (30.7–39.0)	31.8 (27.0–36.5)	0.31
Men Handgrip strength (kgf)	41.2 (34.5–47.9)	41.2 (38.9–43.4)	0.98
Women Handgrip strength (kgf)	29.6 (26.4–32.8)	24.1 (19.6–28.6)	0.05
Medicines			
Antihypertensives	9 (45%)	9 (45%)	
Statins	1 (5%)	3 (15%)	
Oral antidiabetics	0 (0%)	1 (5%)	
Anticoagulant	1 (5%)	0 (0%)	
Antirheumatics	0 (0%)	1 (5%)	
Antidepressants	1 (5%)	0 (0%)	
None	10 (50%)	11 (55%)	

Values are means (95% CI), number, and percentage. NSG: Nonsarcopenic group. SG: Sarcopenic group. BMI: Body Mass Index, BF: Body Fat, RSMI: Relative Skeletal Muscle Mass Index, SPPB: Short Physical Performance Battery, STS: Sit-to-Stand test. Bold values denote statistical significance at the *p* < 0.05 level.

**Table 2:** Comparison of hemodynamic parameters between the interventions in the nonsarcopenic and the sarcopenic group.

Outcomes	Intervention	NSG	SG
Δ HR, bpm	Without WBV	1.80 (−0.82–4.43)	3.50 (0.90–6.2)
	WBV	6.30 (3.68–8.92)	7.10 (4.44–9.70)
	Mean Difference	4.50 (0.78–8.20) *	3.50 (−0.177–7.24)
Δ peak HR, bpm	Without WBV	4.90 (2.20–7.60)	7.20 (4.46–9.94)
	WBV	11.60 (8.86–14.34)	13.55 (10.81–16.30)
	Mean Difference	6.70 (2.83–10.57) *	6.35 (2.47–10.22) *
Δ SBP, mmHg	Without WBV	−2.00 (−6.54–2.54)	−1.00 (−5.54–3.54)
	WBV	4.00 (−0.54–8.54)	−2.00 (−6.54–2.54)
	Mean Difference	6.00 (−0.42–12.42)	1.00 (−5.42–7.42)
Δ DBP, mmHg	Without WBV	0.00 (−3.50–3.50)	−0.50 (−3.40–2.99)
	WBV	1.50 (−1.99–4.99)	2.00 (−1.50–5.50)
	Mean Difference	1.50 (−3.44–6.44)	2.50 (−2.44–7.44)
Δ MAP, mmHg	Without WBV	0.17 (−4.80–5.13)	1.17 (−3.80–6.13)
	WBV	−10.67 (−15.63–−5.70)	2.66 (−2.30–7.63)
	Mean Difference	−10.83 (−17.85–−3.81) *	−1.50 (−8.52–−5.52)
Δ DP, mmHg, bpm	Without WBV	110.60 (−370.36 −591.56)	365.90 (−115.06–846.86)
	WBV	1160.75 (679.73–1641.70)	822.85 (341.90–1303.80)
	Mean Difference	1050.15 (369.97–1730.33) *	456.95 (−223.23–1137.13)
	Without WBV	0.00 (−0.05–0.05)	0.00 (−0.056–0.056)
Δ SPE, points	WBV	0.05 (−0.006–0.106)	0.02 (−0.03–0.08)
	Mean Difference	−0.05 (-0.13–0.03)	−0.02 (−0.10–0.05)

Values are means (95% CI). HR: heart rate; peak HR: peak heart rate; systolic blood pressure (SBP); diastolic blood pressure (DBP); MAP: mean arterial pressure; DP: double product; SPE: subjective perception of effort. NSG: Nonsarcopenic group and SG: Sarcopenic group. Experimental design in randomized blocks (between-intervention, within-intervention, interaction analyses). Two-way ANOVA (2 intervention vs. 2 moments), in both groups (nonsarcopenic and sarcopenic). * Post hoc Bonferroni significance.

**Table 3:** Effects and interaction values found by Two-way ANOVA analysis of the data presented in Table 2.

Outcomes	Between Groups (NSG vs. SG)		Within Groups (Without-WBV vs. WBV)		Interaction	
	*p*	F	*p*	F	*p*	F
Δ HR, bpm	0.35	0.89	**0.003**	9.3	0.72	0.13
Δ peak HR, bpm	0.12	2.38	**0.000**	22.51	0.89	0.02
Δ SBP, mmHg	0.28	1.20	0.28	1.20	0.13	2.36
Δ DBP, mmHg	1.00	0.00	0.26	1.30	0.78	0.08
Δ MAP, mmHg	**0.005**	8.27	0.06	3.50	**0.02**	6.12
Δ DP, mmHg, bpm	0.86	0.03	**0.003**	9.74	0.22	1.50
Δ SPE, points	0.65	0.20	0.18	1.80	0.65	0.20

Legend: HR: heart rate; peak HR: peak heart rate; systolic blood pressure (SBP); diastolic blood pressure (DBP); MAP: mean arterial pressure; DP: double product; SPE: subjective perception of effort. NSG: Nonsarcopenic group and SG: Sarcopenic group. Experimental design in randomized blocks (between-intervention, within-intervention, interaction analyses). Two-way ANOVA (2 intervention vs. 2 moments), in both groups (nonsarcopenic and sarcopenic). Bold values denote statistical significance at the *p* < 0.05 level.

## Data Availability

The data presented in this study are available on reasonable request from the corresponding author.
